# Impacts of Dietary Phytochemicals in the Presence and Absence of Pesticides on Longevity of Honey Bees (*Apis mellifera*)

**DOI:** 10.3390/insects8010022

**Published:** 2017-02-14

**Authors:** Ling-Hsiu Liao, Wen-Yen Wu, May R. Berenbaum

**Affiliations:** Department of Entomology, University of Illinois at Urbana-Champaign, 505 S. Goodwin, Urbana, IL 61801-3795, USA; liao19@illinois.edu (L.-H.L.); wywu@illinois.edu (W.-Y.W.)

**Keywords:** *p*-coumaric acid, quercetin, detoxification, lifespan, nutrition, bifenthrin, β-cyfluthrin

## Abstract

Because certain flavonols and phenolic acids are found in pollen and nectar of most angiosperms, they are routinely ingested by *Apis mellifera*, the western honey bee. The flavonol quercetin and the phenolic acid *p*-coumaric acid are known to upregulate detoxification enzymes in adult bees; their presence or absence in the diet may thus affect the toxicity of ingested pesticides. We conducted a series of longevity assays with one-day-old adult workers to test if dietary phytochemicals enhance longevity and pesticide tolerance. One-day-old bees were maintained on sugar syrup with or without casein (a phytochemical-free protein source) in the presence or absence of quercetin and *p*-coumaric acid as well as in the presence or absence of two pyrethroid insecticides, bifenthrin and β-cyfluthrin. Dietary quercetin (hazard ratio, HR = 0.82), *p*-coumaric acid (HR = 0.91) and casein (HR = 0.74) were associated with extended lifespan and the two pyrethroid insecticides, 4 ppm bifenthrin (HR = 9.17) and 0.5 ppm β-cyfluthrin (HR = 1.34), reduced lifespan. Dietary quercetin enhanced tolerance of both pyrethroids; *p*-coumaric acid had a similar effect trend, although of reduced magnitude. Casein in the diet appears to eliminate the life-prolonging effect of *p*-coumaric acid in the absence of quercetin. Collectively, these assays demonstrate that dietary phytochemicals influence honey bee longevity and pesticide stress; substituting sugar syrups for honey or yeast/soy flour patties may thus have hitherto unrecognized impacts on adult bee health.

## 1. Introduction

Nectar and pollen, both raw and in their processed forms as honey and beebread, have long been considered as the principal natural sources of carbohydrate and protein, respectively, for honey bees. Contemporary beekeeping practices have led to the creation of substitutes or supplements for honey and pollen, notably sucrose or fructose for honey and soy flour diet for pollen [1]. However, phytochemicals clearly serve important functions beyond carbohydrate and protein nutrition for honey bees [2,3,4] and their absence from dietary supplements or substitutes may have effects on honey bees that are as yet undetermined.

Among the phytochemicals present in most pollens and in honey from a diversity of nectar sources, the phenolic acid *p*-coumaric acid and the flavonol quercetin upon ingestion upregulate expression of a diversity of xenobiotic-metabolizing cytochrome P450 genes, including those encoding CYP9Q enzymes, in both adults and larvae [5,6]. When heterologously expressed in a baculovirus expression system, three members of the CYP9Q subfamily upregulated by quercetin, CYP9Q1, CYP9Q2, and CYP9Q3, metabolize quercetin as well as pyrethroid and organophosphate pesticides [5]. In bioassays, Johnson et al. [7] showed that quercetin can reduce toxicity of tau-fluvalinate, a broad-spectrum pyrethroid acaricide. Collectively, these findings strongly suggest that honey is more than a fuel source and that pollen is more than merely a protein source for the bees; the phytochemicals of honey and pollen appear to play an essential role in honey bee health, particularly in the presence of pesticides.

In addition to influencing detoxification capacity, phytochemicals may affect the lifespan of bees, as they are known to do in other organisms [8,9]. Quercetin is an inducer of SirT1 [10,11,12], a member of the sirtuin family of proteins, considered as mediators of lifespan extension via the caloric restriction effect in many organisms [13]. Honey bees [14] and *Drosophila melanogaster* [15,16] are among the insects known to exhibit the caloric restriction effect on lifespan [14,17]; almost all known genes in the sirtuin family (i.e., *SirT1*, *Sir2*, *Sir4*, *Sir5*, *Sir6*, and *Sir7*) are represented in the honey bee genome. Other evidence suggests that phytochemicals in honey, pollen and propolis may affect life span. With respect to *p*-coumaric acid, Mao et al. [2] found that rearing larvae in vitro on a royal jelly diet with *p*-coumaric acid reduced ovary development of adult bees. In view of the negative correlation between ovary development and survival rate in adult honey bees [17], this finding suggests that consuming *p*-coumaric acid may increase survival and promote longevity. In other studies, however, greater ovarian development (along with greater vitellogenin production) in adult workers is correlated with increased longevity [18,19]. Thus, predicting the effects of consuming *p*-coumaric acid by adult workers is not straightforward. As for quercetin, some circumstantial evidence links its presence to enhanced longevity in honey bees. Quercetin is a ubiquitous constituent of propolis, a hive sealant derived from plant resins that is typically rich in flavonols and other phenolics. In Brazil, Nicodemo et al. [20] found that honey bee longevity is 6.6% greater in hives with more propolis present; propolis typical of this region has been shown to be rich in quercetin, along with phenolic acids [21].

To characterize the effect of phytochemicals in lifespan of honey bees, we carried out a series of longevity bioassays. One-day-old bees were provided with a sugar-casein protein diet to standardize their nutrition. The sugar-casein protein diet was prepared with and without phytochemicals in four combinations (blank solvent control, *p*-coumaric acid, quercetin, and quercetin plus *p*-coumaric acid) to test the effects of these phytochemicals on longevity of honey bee workers. Because the absence of a queen may induce ovary development and egg-laying in workers, both of which affect longevity, we added a commercial queen pheromone strip to the cages to maintain workers in the sterile state. Moreover, in order to determine whether phytochemicals alter the ability of honey bee workers to detoxify pesticides, we carried out a concurrent series of bioassays in which pesticides were added to the sugar/casein diets. Two pyrethroid insecticides, bifenthrin and β-cyfluthrin, were tested; both have been found as contaminants of wax, pollen, and bee bodies in North American hives [22].

## 2. Materials and Methods

### 2.1. Experimental Insects

The experiment was conducted with western honey bees (*Apis mellifera*) kept in the apiary of the University of Illinois Bee Research Facility at Urbana-Champaign. In June 2016, three frames of capped brood were collected from a single naturally mated queen colony and then incubated in a dark room at 34 °C to obtain newly emerged adult workers. These bees, collected within 24 h of emergence, were introduced into small cages (9 oz/266 mL plastic cup with several ventilation holes and two feeding holes) in groups of 25 individuals. One-tenth of a strip of commercialized artificial queen mandibular pheromone (DC-715, Mann Lake Ltd., Hackensack, MN, USA) was also introduced into each cage at the same time. Cages of newly emerged adult workers were prepared and randomly assigned evenly to three groups, a control and two pesticide treatment groups. The caged bees were immediately provided with water and diet, corresponding to their treatment group. All bees used for this study were collected and prepared within a five-day period. One cage of bees in the control group was accidentally lost, so a total of 119 cages of 25 individual honey bee workers each were tested in this study.

### 2.2. Effects of Dietary Protein, Phytochemicals and Pesticides on Longevity

During the experiment, the caged bees were kept in a dark room at 32.2 °C with 50% relative humility. Each cage was equipped with a water feeder and a 50% (*w*/*v*) sucrose water-based diet feeder. The feeders were made by cutting a hole 6 mm in diameter on the top of a 2 mL micro-centrifuge tube. Bees could access water or food easily through the opening. Water was provided ad libitum; the water feeder was replaced every 5 days or whenever it appeared to be nearly empty. The diet feeders were replaced daily, just after the daily survival check of the caged bees. Approximately 1.5 mL sucrose water-based diet was used to fill each feeder in every cage; this amount was more than sufficient to feed all of the bees in each cage. The assay continued until all test subjects had died.

To determine the effects of pesticides on longevity, three types of amendments were made to the base diet: 4 ppm bifenthrin (N-11203, ChemService, Inc., West Chester, PA, USA), 0.5 ppm β-cyfluthrin (N-11191, ChemService, Inc., West Chester, PA, USA) and no amendments (control). The concentrations used for the tested pesticides concentration were based on pilot bioassays [23]. Within each pesticide treatment, two base diets were compared: protein-rich (protein:carbohydrate = 1:12, [17]) and protein-free. Casein, an animal-derived protein product free from phytochemicals, was used here as a supplemental protein supplement, as it has been used in many insect artificial diets [24]. Within each base diet, phytochemical amendments were compared; these amendments included 0.5 mM *p*-coumaric acid (C9008, Sigma-Aldrich Co. LLC., St. Louis, MO, USA) (PC), 0.25 mM quercetin (Q4951, Sigma-Aldrich Co. LLC., St. Louis, MO, USA) treatment (Qc), 0.5 mM *p*-coumaric acid and 0.25 mM quercetin-combined (PQ), and no phytochemicals (Control, CD). The phytochemical concentrations selected for testing in this work, also used in previous studies [2,25], were designed to fit within the range of concentrations ingested by worker bees over the course of their adult lives. Phytochemical concentrations vary in honey bee diets depending on plant species, tissue type, season, and geographic locality. Yet another source of variation is the age- and task-related polyethism of adult bees, whereby tasks in the hive are associated with different nutritional demands as bees age or colony conditions change. The concentrations tested are within the natural range documented in honey, pollen and beebread, the three primary sources of ingested phytochemicals. The concentration of *p*-coumaric acid used, 0.5 mM (82 μg/g), is within the range of concentrations in local honey and beebread [2] and our tested concentration of quercetin (0.25 mM = 75.6 mg/kg), although higher than concentrations typically encountered in honey (up to 4.86 mg/kg) [26], is lower than concentrations in pollen (up to 529.8 mg/kg) [27,28] and in beebread (495.8 mg/kg) [29]. There were 24 different treatments in each experimental replicate, and each treatment had five replicates (Table 1).

The protein-rich (casein^+^) stock syrup was prepared by adding 25 g casein (C3400, Sigma-Aldrich Co. LLC., St. Louis, MO, USA) into 600 g 50% (*w*/*v*) sucrose water. The pesticides and phytochemicals were first dissolved in dimethyl sulfoxide (DMSO; D128, Fisher Scientific International, Inc., Pittsburgh, PA, USA) to make the 400× concentrated stock solutions. Finally, the sucrose water-based diets were prepared by adding 0.125 mL 400× phytochemical stock solutions into protein-free (casein^−^) or protein-rich (casein^+^) 50% syrups to make a total volume of 50 mL. In addition, the unamended phytochemical-free control diet was prepared by adding 0.125 mL DMSO to protein-free or protein-rich 50% syrup for a volume of 50 mL. As the result, all of the diets contained equal amounts of 0.25% DMSO.

### 2.3. Effects of Dietary Protein, Phytochemicals and Pesticides on Diet Consumption

Diet feeders were weighed individually before and after being made available to the bees to measure daily diet consumption. One additional cage, designated the evaporation cage, was established for each treatment in order to correct estimates of diet consumption for losses due to evaporation. The feeders in the evaporation cage were filled with the same tested syrup, to estimate as precisely as possible the evaporative characteristics of the diets being tested. The corrected weight loss of each feeder was then divided by the number of surviving bees in each cage to calculate diet consumption per bee per day.

### 2.4. Statistics

Statistical analyses were conducted using OriginPro 2016 software (OriginLab Corporation, Northampton, MA, USA) and SPSS software (version 22.0; IBM Corp., Armonk, NY, USA). The effects of treatment factors on bee survival were analyzed using the Cox proportional hazards model. When an effect of treatment was significant in the Cox model, the hazard ratio was calculated to express the magnitude of the effect of treatment. The relationship between the hazard ratio (HR) and the survival function can be shown as follows:

S_t_(*t*) = S_c_(*t*)^HR^,

where S_t_(*t*) is survival (value between 0 and 1) of treatment group at time *t* and S_c_(*t*) is the survival of control group at the same time *t* [30]. Therefore, when HR > 1, the survival of the treatment group is lower than that of the control group, which means that the treatment factor presents a high risk, and, when HR < 1, the survival of the treatment group is higher than that of the control group, which means that the treatment factor reduces the hazard risk.

In addition, survival curves for each treatment group were obtained through the Kaplan-Meier estimator, and the differences between the curves were compared by the log-rank test with Bonferroni correction. The differences in the daily consumption per bee between treatment groups were analyzed by Kruskal-Wallis analysis of variance (ANOVA) and the post-hoc comparisons were performed by Mann-Whitney *u*-test with Bonferroni correction.

## 3. Results

### 3.1. Effects of Dietary Protein, Phytochemicals and Pesticides on Survival

**Figure 1:**
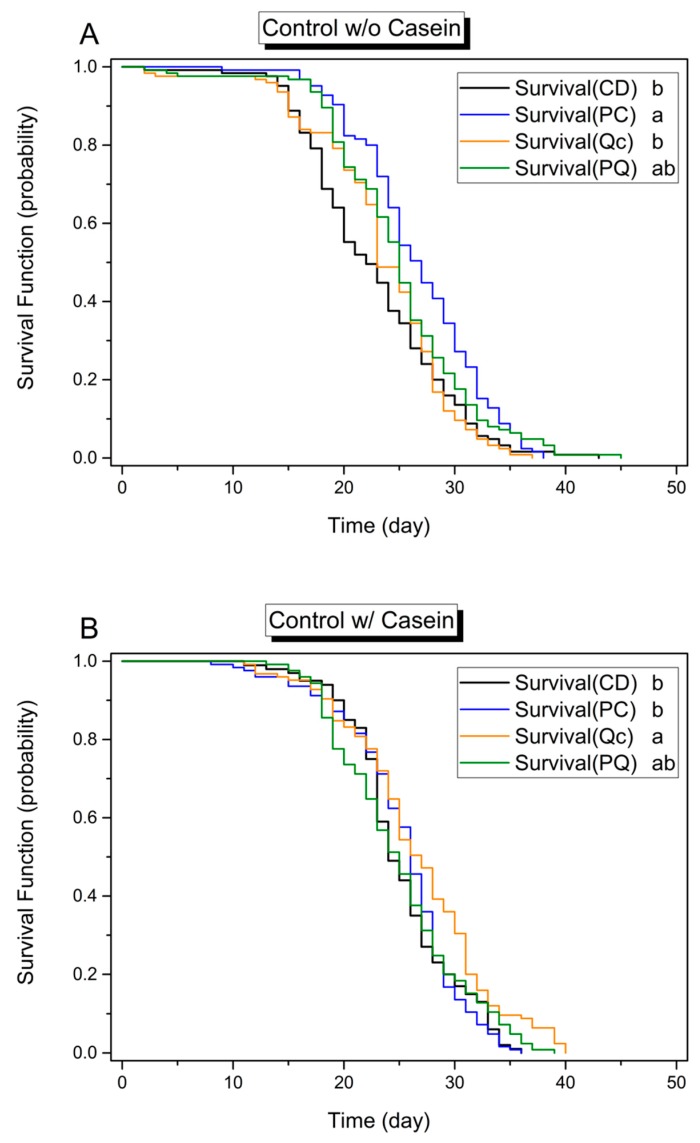
Kaplan–Meier plot of honey bee survival function on different diets with different phytochemical supplements. These diets were (**A**) protein-free or (**B**) protein-supplemented. CD, diet lacking phytochemicals; PC, diet containing 0.5 mM *p*-coumaric acid; Qc, diet containing 0.25 mM quercetin; PQ, diet containing 0.5 mM *p*-coumaric acid and 0.25 mM quercetin. (*n* = 100 for protein-rich and phytochemical-free diet group (CD in Figure 1B), and *n* = 125 for the other groups.) Different lower-case letters indicate statistical differences between treatments (log-rank paired test, *p* < 0.0083 after Bonferroni correction).

**Figure 2:**
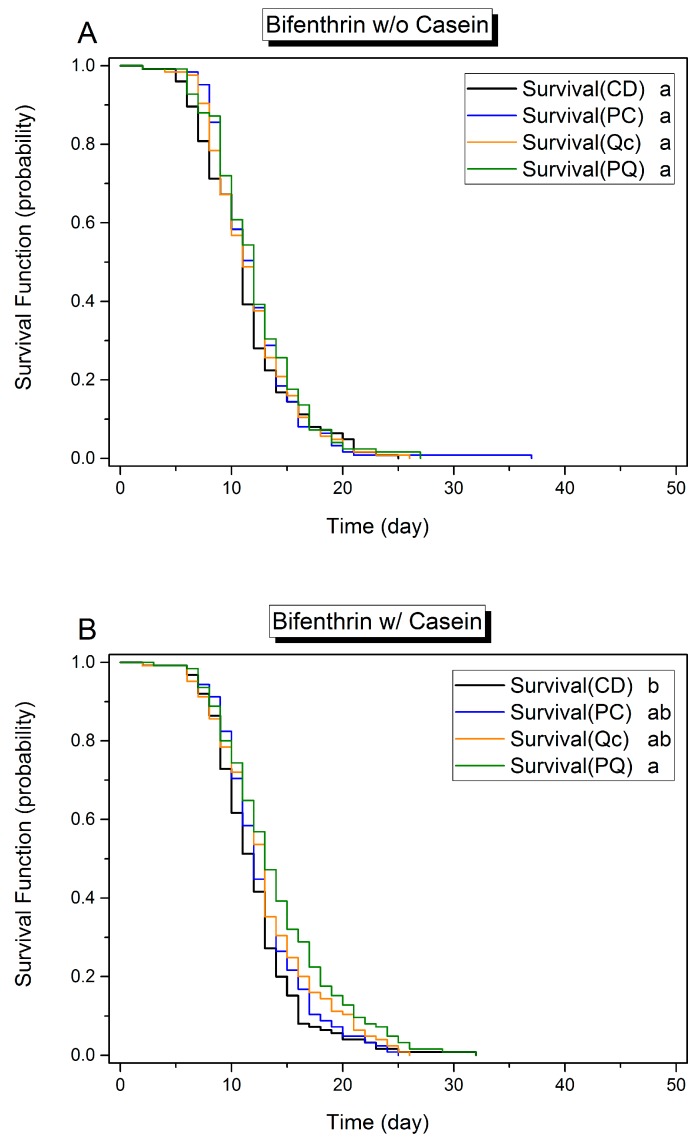
Kaplan–Meier plot of honey bee survival function on different diets with different phytochemical supplements and bifenthrin amendment. Theses diets were (**A**) protein-free or (**B**) protein-supplemented. CD, diet lacking phytochemicals; PC, diet containing 0.5 mM *p*-coumaric acid; Qc, diet containing 0.25 mM quercetin; PQ, diet containing 0.5 mM *p*-coumaric acid and 0.25 mM quercetin. (*n* = 125 for each group.) Different lower-case letters indicate statistical differences between treatments (log-rank paired test, *p* < 0.0083 after Bonferroni correction).

**Figure 3:**
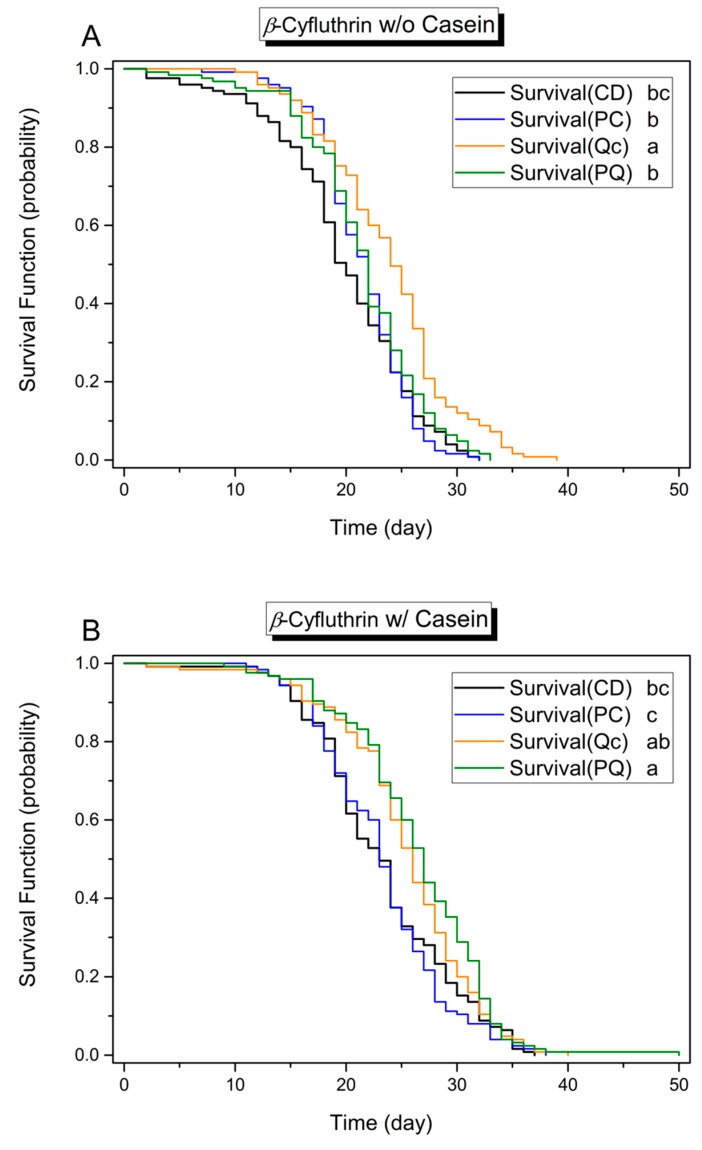
Kaplan–Meier plot of honey bee survival function on diets with different phytochemical supplements and β-cyfluthrin amendment. These diets were (**A**) protein-free or (**B**) protein-supplemented. CD, diet lacking phytochemicals; PC, diet containing 0.5 mM *p*-coumaric acid; Qc, diet containing 0.25 mM quercetin; PQ, diet containing 0.5 mM *p*-coumaric acid and 0.25 mM quercetin. (*n* = 125 for each group.) Different lower-case letters indicate statistical differences between treatments (log-rank paired test, *p* < 0.0083 after Bonferroni correction).

By the Cox proportional hazards models (Cox model) analysis on the pooled results of 2975 caged honey bees, the survival analysis revealed that all tested experimental factors (protein, phytochemicals, and pesticides) affected the longevity of adult bees (Table 2 and Table 3). Overall, according to the hazard ratio of the Cox model, as compared to controls, treatment diets affected longevity at different levels and in the order casein > quercetin > *p*-coumaric acid > 0.5 ppm β-cyfluthrin > 4 ppm bifenthrin. Casein (HR = 0.74, *χ*^2^ = 66.31, *p* < 0.001), quercetin (HR = 0.82, *χ*^2^ = 27.93, *p* < 0.01), and *p*-coumaric acid (HR = 0.91, *χ*^2^ = 5.93, *p* = 0.015) positively influenced longevity of bees. The effects of dietary protein and phytochemicals were mirrored in the Kaplan–Meier survival plot. For example, the survival curves plotted in Figure 1, Figure 2 and Figure 3 in all panels marked A, representing protein-free treatments, are generally shifted leftward (reduced longevity) relative to those in all panels marked B, representing protein-rich treatments. Similarly, across Figure 1, Figure 2 and Figure 3, plots of treatments including phytochemicals, depicted in blue, orange, or green, are generally shifted rightward (toward enhanced longevity) relative to the (black) control group curves (Figure 1, Figure 2 and Figure 3).

In contrast, the two pyrethroid pesticides, bifenthrin and β-cyfluthrin, negatively affected worker longevity. Bifenthrin and β-cyfluthrin diets yielded higher hazard ratios of 9.17 and 1.35 (*χ*^2^ = 1741.640, *p* < 0.001 and *χ*^2^ = 42.157, *p* < 0.001, respectively) and as well reduced mean survival time by 12.63 and 1.89 days (−50.5% and −7.5%), respectively, for the tested bees.

### 3.2. Effect of Phytochemicals on Longevity in the Absence of Pesticides

In the absence of pesticides, the Cox model analysis revealed that protein level and phytochemicals did not affect the longevity of adult bees. However, by making the PQ treatment an independent covariance factor in the Cox model, all phytochemical treatments (PC, Qc, and PQ) enhanced the longevity of bees (*n* = 975, Cox model, PC: *χ*^2^ = 9.76, *p* = 0.002 < 0.01, hazard ratio = 0.75; Qc: *χ*^2^ = 5.70, *p* = 0.017 < 0.05, HR = 0.80; PQ: *χ*^2^ = 4.18, *p* = 0.04 < 0.05, HR = 0.83; PQ treatment as an independent covariance factor) but not the protein treatment (*χ*^2^ = 3.07, *p* = 0.08). The results suggest that *p*-coumaric acid and quercetin together may have some synergistic effects. Regarding the presence or absence of protein, in the absence of pesticide stress, while casein supplementation did not extend longevity of bees by Cox model analysis, the cross-comparisons still show that casein supplementation enhanced longevity by 13.9% (+3.26 days) in the Qc subgroup (treatment 3 vs. 7, log rank test with Bonferroni correction, *χ*^2^ = 19.16, *p* < 0.002).

*p*-Coumaric acid (Cox model, HR = 0.68, *χ*^2^ = 17.684, *p* < 0.001, PQ not an independent covariance factor) enhanced the longevity of bees by 17.6% (+4.00 days; CD vs. PC, log-rank test with Bonferroni correction, *χ*^2^ = 17.275, *p* < 0.008; Tables S1 and S2), when they fed on the casein-free pesticide-free diet (*n* = 500) (Figure 1A). In contrast, bees on pesticide-free casein-containing diet experienced greater longevity enhancement relative to the control diet with quercetin in the diet (CD vs. Qc, 6.2% longer (+1.55 days), log-rank test, *χ*^2^ = 7.444, *p* = 0.006 < 0.0083 after Bonferroni correction; Figure 1B). However, bees on diets containing both quercetin and *p*-coumaric acid did not experience longevity enhancement relative to those in the control treatment or in the *p*-coumaric acid treatment. Thus, while 0.25 mM quercetin in casein diet enhanced longevity of the caged bees, adding 0.5 mM *p*-coumaric acid may diminish the benefit of quercetin in a protein-rich diet.

### 3.3. Effect of Phytochemicals on Survival in the Presence of Pesticides

Casein supplementation improved the survival of caged honey bees in the presence of either pyrethroid insecticide—on diets with 4 ppm bifenthrin by 11.0% (+1.29 days longer; *n* = 1000; Cox model, *χ*^2^ = 16.553, *p* < 0.001, HR = 0.77) and on diets with 0.5 ppm β-cyfluthrin by 14.2% (+3.06 days longer; *n* = 1000; Cox model, *χ*^2^ = 68.787, *p* < 0.001, HR = 0.58) (Table 3 and Table S1). With respect to phytochemicals, quercetin prolonged survival on the bifenthrin-containing casein diets by 9.1% (+1.10 days longer; Cox model, *χ*^2^ = 8.704, *p* = 0.003, HR = 0.76), on the β-cyfluthrin-containing casein-free diets by 20.6% (+4.07 days longer; *χ*^2^ = 8.704, *p* = 0.003, HR = 0.67) and on the β-cyfluthrin-containing casein diets by 9.2% (+2.15 days longer; *χ*^2^ = 16.603, *p* < 0.001, HR = 0.69). However, the contribution of *p*-coumaric acid to lifespan enhancement is not clear. Its presence appears to have a positive effect on bifenthrin-treated bees (Cox model, *χ*^2^ = 4.0318, *p* = 0.0447 < 0.05, HR = 0.88), but, upon further analysis of subgroups, *p*-coumaric acid alone in the diet contributes only a slightly positive trend that does not reach statistical significance. Moreover, while the PQ treatment extended bee survival on the bifenthrin-containing casein diets by 16.6% (+2.01 days longer, log rank test, *χ*^2^ = 11.826, *p* < 0.0018 after Bonferroni correction) and on the β-cyfluthrin-containing casein diets by 13.6% (+3.18 days longer, log rank test, *χ*^2^ = 9.979, *p* < 0.0018 after Bonferroni correction), longevity of individuals on diets containing *p*-coumaric acid or quercetin alone diet was not enhanced (Table S2).

On casein-free diets containing bifenthrin, phytochemical treatments had no significant effect on the survival curves (Figure 2A). In contrast, on casein-supplemented diets containing bifenthrin, individuals on diets also containing both phytochemicals (PQ subgroup) experienced enhanced longevity over their control subgroup (CD, casein^+^, bifenthrin^+^ vs. PQ, casein^+^, bifenthrin^+^, log rank test, *χ*^2^ = 11.826, *p* < 0.0083 after Bonferroni correction) by 16.6% (+2.01 days (Figure 2B). In contrast, individuals on diets containing one but not both phytochemicals did not experience a change in longevity.

Aside from the β-cyfluthrin-containing casein-free diet, bees in Qc treatments experienced the greatest longevity enhancement, living 20.6% longer (+4.07 days) than those in the control treatment (log rank test, *χ*^2^ = 26.704, *p* < 0.0083 after Bonferroni correction) (Figure 3A). However, diets containing both phytochemicals reduced survival relative to diets containing quercetin alone, causing a 9.7% reduction in lifespan (−2.13 days; log rank test, *χ*^2^ = 13.020, *p* < 0.0083 after Bonferroni correction) while there was no significant lifespan difference in longevity between PQ and PC treatment diets (Figure 3), again demonstrating the adverse effects of the combination of *p*-coumaric acid and quercetin in a diet. With respect to the β-cyfluthrin-containing casein diets, bees on the diet containing both phytochemicals experienced greater longevity relative to bees on control diets (13.6% longer, +3.18 days; log rank test, *χ*^2^ = 9.979, *p* < 0.0083 after Bonferroni correction) or diets containing *p*-coumaric acid alone (14.1% longer lifespan, +3.29 days; log rank test, *χ*^2^ = 16.272, *p* < 0.0083 after Bonferroni correction) as well (Figure 3B).

Cross-comparisons between casein-free and casein-supplemented treatments in the presence of β-cyfluthrin revealed that consuming diets supplemented with PC, PQ and casein enhanced bee longevity to an even greater extent [casein^+^, β-cyfluthrin^+^ vs. casein^−^ β-cyfluthrin^+^, log-rank test with Bonferroni correction, CD: 18.6% longer lifespan (+3.54 days), *χ^2^* = 21.74, *p* < 0.0018; PC: 8.5% longer lifespan (+1.82 days), *χ*^2^ = 14.44, *p* < 0.0018; PQ: 23.5% longer lifespan (+5.06 days), *χ*^2^ = 52.49, *p* < 0.0018]. Moreover, quercetin may reduce β-cyfluthrin toxicity; bees consuming diets containing both quercetin and β-cyfluthrin survived as well as those consuming unamended diets (Tables S1 and S2).

### 3.4. Effects of Dietary Protein, Phytochemicals and Pesticides on Diet Consumption

**Figure 4:**
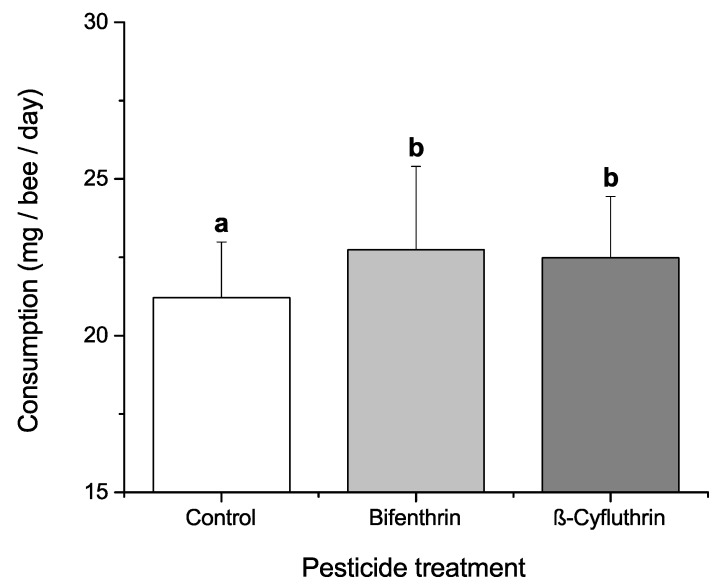
Mean + SD milligrams syrup diet consumption per bee per day over entire adult lifespan in cages containing different pesticide treatments. Different lower-case letters indicate significant differences (Kruskal-Wallis ANOVA, *χ*^2^ = 10.255, *p* = 0.006; post-hoc comparisons by Mann-Whitney *U*-test with Bonferroni correction, adjusted *alpha* = 0.017).

By Kruskal-Wallis ANOVA, pesticide amendment was the only factor with a significant effect (*χ*^2^ = 10.255, *p* = 0.006) on daily diet consumption per bee. Bees consuming the control diets lacking pesticides ingested less diet than did bees consuming diets containing bifenthrin (Mann-Whitney *U* = 483, *n*_control_ = 39, *n*_bifenthrin_ = 40, *p* = 0.004) or β-cyfluthrin (Mann-Whitney *U* = 488, *n*_control_ = 39, *n*_β-cyfluthrin_ = 40, *p* = 0.004; there was no significant difference between the two pesticide treatments (Mann-Whitney *U* = 801, *n*_bifenthrin_ = *n*_β-cyfluthrin_ = 40, *p* = 0.996) (Figure 4). All other treatment factors or other subgroup combinations (e.g., phytochemical amendments to casein-free diet) had no significant effect on daily syrup consumption.

## 4. Discussion

Overall, the presence of a dietary protein prolongs the longevity of adult honey bees and the presence of pyrethroid insecticides in the diet reduces the longevity of honey bees. These findings are entirely consistent with past research [1] and in and of themselves are not novel, although the ingestion-enhancing effect of both bifenthrin and β-cyfluthrin has not been widely documented previously. What is novel, however, is the finding that two phytochemicals that are ubiquitous in the natural diet of honey bees can enhance longevity, despite the fact that they are not known to provide any strictly nutritional benefit. Quercetin ingestion also significantly enhanced tolerance of pyrethroids (bifenthrin, and beta-cyfluthrin) and survival rate of workers, as previously documented [7].

In honey bees, quercetin is known to upregulate detoxification genes, including *CYP9Q* genes that detoxify pyrethroid pesticides [5,6], and this upregulation may account for the protective effect of quercetin against pesticides observed in this study. As well, as a powerful antioxidant [31], quercetin may reduce the toxic effects of pyrethroids by ameliorating the oxidative stress caused by pyrethroid pesticides [32,33]. With respect to longevity enhancement, in addition to its antioxidative properties, quercetin in honey bees may influence expression of potential longevity genes (sirtuin family), as it does in mammals [10,11,12], or expression of antioxidant enzymes associated with longevity, as it does in other plant-feeding insects [34].

With respect to ameliorating pesticide toxicity, quercetin differs in its impact depending on the identity of the pesticide. On diets containing bifenthrin, quercetin yields a hazard ratio of 0.85 but the hazard ratio is only 0.67 in β-cyfluthrin treatments. This apparent difference in efficacy may be a function of pesticide concentrations used in this study rather than toxicity per se. The LC_50_ value for bifenthrin is 17 ppm as reported by Dai et al. [35], which contrasts with the LD_50_ value of 15 ng/bee reported by Mullin et al. [22]; in this study, bifenthrin was used at a concentration of 4 ppm (approximate 90.96 ng/bee/day). In contrast, β-cyfluthrin has a reported LD_50_ of 22 ng/bee [22]; β-cyfluthrin was used in this study at a concentration of 0.5 ppm (approximate 11.244 ng/bee/day). At this low level of stress, quercetin alone could rescue longevity even in the absence of dietary protein amendment.

Although *p*-coumaric acid amendment appeared to ameliorate effects of bifenthrin ingestion on longevity at the tested concentrations, the trend is not statistically significant. Notably, *p*-coumaric acid added to a diet lacking both casein and pesticide does significantly enhance longevity (see Figure 1A). In diets containing casein, however, the presence of *p*-coumaric acid may reduce longevity, although any such effect appears to be subtle. The mechanism underlying this apparent antagonism is open to speculation; given the fact that *p*-coumaric acid upregulates a diversity of protein-encoding genes [3], its presence in the diet may alter protein utilization rates. Similarly, whereas diets lacking both pesticides and casein promote greater longevity when amended with both phytochemicals together, this effect of phytochemical amendment is not observed in diets containing casein. In contrast, when pesticides are present in the diet, bees consuming diets containing both phytochemicals together generally experience greater longevity relative to bees on control diets.

## 5. Conclusions

In this study, *p*-coumaric acid and quercetin, ubiquitous phytochemicals in the natural diet of honey bees, generally have a beneficial effect on honey bee longevity, most dramatically in the case of dietary quercetin in the presence of two pyrethroid insecticides. Previous studies have shown that, in honey bees, malnutrition can increase sensitivity to pesticides [36], reduce immunocompetence [37] and alter gene expression in protein metabolism and oxidation-reduction in fat body [38]. Together, these findings suggest that substituting sugar syrups for honey or yeast/soy flour patties for pollen may not only cause malnutrition but may also have unanticipated effects on lifespan in the presence of environmental stressors. Notwithstanding, there is enough evidence of antagonistic interactions or negative effects that simply augmenting honey bee sugar substitutes or soy flour substitutes with phytochemicals ad libitum is inadvisable without additional information on the mechanisms by which phytochemicals can enhance longevity or ameliorate pesticide toxicity. The complexity of the social organization of honey bee colonies means that these phytochemicals may have effects that operate only at the colony level. Mao et al. [2], e.g., reported that *p*-coumaric acid can alter expression of caste determination genes and Gao et al. [39] found high concentrations of quercetin in diets may boost worker resistance to queen signals in the hive and lead to the production of laying workers. Clearly, these two phytochemicals, and possibly other widely distributed constituents of pollens, nectars and propolis, have non-nutritive impacts on honey bee health that reflect the long evolutionary association between honey bees and flowering plants.

## Figures and Tables

**Table 1:** Summary of 24 different treatments.

Protein		Protein-free (casein^−^)				Protein-rich (casein^+^)			
**Phytochemical**		Control (CD)	0.5 mM	0.25 mM quercetin (Qc)	0.5 mM	Control (CD)	0.5 mM	0.25 mM quercetin (Qc)	0.5 mM
			*p*-coumaric acid (PC)		*p*-coumaric acid and 0.25 mM quercetin (PQ)		*p*-coumaric acid (PC)		*p*-coumaric acid and 0.25 mM quercetin (PQ)
**Pesticide**	**Pesticide-free**	Treatment 1	Treatment 2	Treatment 3	Treatment 4	Treatment 5	Treatment 6	Treatment 7	Treatment 8
	**β-Cyfluthrin**	Treatment 9	Treatment 10	Treatment 11	Treatment 12	Treatment 13	Treatment 14	Treatment 15	Treatment 16
	**Bifenthrin**	Treatment 17	Treatment 18	Treatment 19	Treatment 20	Treatment 21	Treatment 22	Treatment 23	Treatment 24

**Table 2:** Cox proportional hazards model analysis of effects of diet amendments on adult honey bee longevity.

Experimental factor	df	Estimate	Standard Error	*χ*^2^	*p*	Hazard Ratio
Casein	1	−0.30	0.04	66.31	< 0.001	0.739 ***
Quercetin	1	−0.20	0.04	27.93	< 0.001	0.823 ***
*p*-Coumaric acid	1	−0.09	0.04	5.93	0.015	0.914 *
β-cyfluthrin	1	0.30	0.05	42.16	< 0.001	1.345 ***
Bifenthrin	1	2.22	0.05	1741.64	< 0.001	9.171 ***

All tested experimental factors (casein, phytochemicals, and pesticides) affected the longevity of the honey bees. Casein, *p*-coumaric acid, and quercetin had positive effects on caged honey bee worker longevity (with hazard ratios < 1). Two pyrethroid insecticides, bifenthrin and β-cyfluthrin, had negative effects on worker longevity (with hazard ratios > 1). *n* = 2,975 caged bees; * *p* < 0.05; *** *p* < 0.001.

**Table 3:** Summary of lifespan comparisons among honey bee workers consuming different diets by the evaluation of Cox proportional hazards model.

	Protein-free (casein^−^)		Protein-rich (casein^+^)
Overall		<^a^	
			
Pesticide-free diet^b^	Treatment 1–4	=	Treatment 5–8
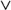			
β-cyfluthrin diet	Treatment 9–12	<	Treatment 13–16
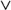			
Bifenthrin diet	Treatment 17–20	<	Treatment 21–24

^a^ The comparison symbols indicate the results obtained by Cox proportional hazards model. (‘<‘ or ‘>‘ was regarded as statistically significant, *p* < 0.05; ‘=‘ indicates no significant difference); ^b^ Pesticide treatment: Pesticide-free, 4 ppm bifenthrin or 0.5 ppm β-cyfluthrin; Casein treatment: casein-free, protein:carbohydrate = 0:1; casein-supplemented, protein:carbohydrate = 1:12.

## Supplementary Materials

The following are available online at http://www.mdpi.com/2075-4450/8/1/22/s1.
