# Supplementary material for: Impacts of Dietary Phytochemicals in the Presence and Absence of Pesticides on Longevity of Honey Bees (Apis mellifera)
Source: Insects. 2017 Feb 14;8(1):22. doi: 10.3390/insects8010022 (PMC5371950; doi:10.3390/insects8010022)
Supplement: Supplementary file 1 [file insects-08-00022-s001.pdf]

Supplementary Materials: Impacts of Dietary Phytochemicals in the Presence and Absence of Pesticides on Longevity of Honey Bees (*Apis mellifera*)

Ling-Hsiu Liao, Wen-Yen Wu and May R. Berenbaum *

Table S1. Summary of the mean and median survival time of all treatments.

Pesticide	Protein	Phytochemical	Mean				Median			
			Estimate	Std. Error	95% Confidence Interval		Estimate	Std. Error	95% Confidence Interval	
					Lower Bound	Upper Bound			Lower Bound	Upper Bound
Pesticide-free	Protein-free	CD	22.72	0.58	21.59	23.85	22.00	1.29	19.47	24.53
		PC	26.72	0.50	25.73	27.71	27.00	0.70	25.64	28.36
		Qc	23.48	0.56	22.39	24.57	23.00	0.60	21.83	24.17
		PQ	25.00	0.59	23.85	26.15	25.00	0.43	24.16	25.84
	Protein-rich	CD	25.19	0.50	24.21	26.17	24.00	0.67	22.69	25.31
		PC	25.33	0.48	24.40	26.26	26.00	0.41	25.19	26.81
		Qc	26.74	0.58	25.62	27.87	27.00	0.70	25.63	28.37
		PQ	24.99	0.51	23.99	25.99	25.00	0.70	23.64	26.36
Bifenthrin	Protein-free	CD	11.24	0.37	10.51	11.97	11.00	0.23	10.55	11.45
		PC	11.95	0.36	11.25	12.66	12.00	0.44	11.15	12.85
		Qc	11.70	0.35	11.03	12.38	11.00	0.47	10.09	11.91
		PQ	12.10	0.37	11.39	12.82	12.00	0.40	11.21	12.79
	Protein-rich	CD	12.09	0.37	11.37	12.81	12.00	0.44	11.14	12.86
		PC	12.79	0.36	12.09	13.49	12.00	0.33	11.36	12.64
		Qc	13.18	0.42	12.37	14.00	13.00	0.29	12.43	13.57
		PQ	14.10	0.47	13.18	15.01	13.00	0.51	12.01	14.00
β-cyfluthrin	Protein-free	CD	19.72	0.56	18.63	20.81	20.00	0.64	18.74	21.26
		PC	21.43	0.36	20.72	22.15	22.00	0.58	20.86	23.14
		Qc	23.79	0.52	22.77	24.82	24.00	0.62	22.78	25.22
		PQ	21.48	0.49	20.51	22.45	22.00	0.40	21.21	22.79
	Protein-rich	CD	23.36	0.57	22.25	24.47	23.00	0.76	21.51	24.49
		PC	23.25	0.50	22.27	24.22	23.00	0.40	22.22	23.78
		Qc	25.51	0.55	24.43	26.59	26.00	0.56	24.91	27.09
		PQ	26.54	0.55	25.45	27.62	27.00	0.56	25.91	28.09

Table S2. Summary of the log-rank paired test of all treatment. Statistically significant differences between 24 treatments are reported in red ($p < 0.0001812$ after Bonferroni correction).

			Pesticide-free															
			Protein free								Protein-rich							
			CD		PC		Qc		PQ		CD		PC		Qc		PQ	
			Chi-Square	Sig.	Chi-Square	Sig.	Chi-Square	Sig.	Chi-Square	Sig.	Chi-Square	Sig.	Chi-Square	Sig.	Chi-Square	Sig.	Chi-Square	Sig.
Pesticide-free	Protein-free	CD			17.275	0.000	0.277	0.599	6.040	0.014	3.996	0.046	4.894	0.027	18.631	0.000	4.861	0.027
		PC	17.275	0.000			17.767	0.000	2.201	0.138	6.613	0.010	7.097	0.008	0.726	0.394	3.822	0.051
		Qc	0.277	0.599	17.767	0.000			3.946	0.047	1.890	0.169	3.440	0.064	19.162	0.000	3.167	0.075
		PQ	6.040	0.014	2.201	0.138	3.946	0.047			0.285	0.594	0.059	0.808	4.728	0.030	0.205	0.651
	Protein-rich	CD	3.996	0.046	6.613	0.010	1.890	0.169	0.285	0.594			0.131	0.717	7.444	0.006	0.236	0.627
		PC	4.894	0.027	7.097	0.008	3.440	0.064	0.059	0.808	0.131	0.717			8.028	0.005	0.048	0.827
		Qc	18.631	0.000	0.726	0.394	19.162	0.000	4.728	0.030	7.444	0.006	8.028	0.005			6.911	0.009
		PQ	4.861	0.027	3.822	0.051	3.167	0.075	0.205	0.651	0.236	0.627	0.048	0.827	6.911	0.009		
Bifenthrin	Protein-free	CD	188.529	0.000	255.248	0.000	206.948	0.000	227.625	0.000	209.306	0.000	240.113	0.000	246.037	0.000	238.633	0.000
		PC	173.742	0.000	228.053	0.000	180.733	0.000	212.527	0.000	180.625	0.000	200.397	0.000	224.427	0.000	213.746	0.000
		Qc	190.707	0.000	258.405	0.000	204.962	0.000	231.681	0.000	208.857	0.000	238.245	0.000	246.216	0.000	241.797	0.000
		PQ	175.647	0.000	246.629	0.000	188.683	0.000	217.518	0.000	193.196	0.000	222.347	0.000	233.984	0.000	225.624	0.000
	Protein-rich	CD	170.381	0.000	234.745	0.000	178.455	0.000	207.531	0.000	185.856	0.000	210.643	0.000	223.490	0.000	217.239	0.000
		PC	164.651	0.000	243.088	0.000	185.881	0.000	215.057	0.000	192.487	0.000	223.472	0.000	230.501	0.000	219.778	0.000
		Qc	137.677	0.000	221.911	0.000	165.130	0.000	187.950	0.000	172.493	0.000	204.542	0.000	209.595	0.000	191.772	0.000
		PQ	100.911	0.000	184.605	0.000	123.719	0.000	148.817	0.000	137.035	0.000	161.619	0.000	175.921	0.000	151.442	0.000
β-cyfluthrin	Protein-free	CD	14.541	0.000	75.788	0.000	25.870	0.000	42.261	0.000	40.126	0.000	52.899	0.000	73.267	0.000	40.470	0.000
		PC	10.471	0.001	75.760	0.000	25.998	0.000	40.075	0.000	39.183	0.000	58.840	0.000	72.461	0.000	36.938	0.000
		Qc	0.801	0.371	11.972	0.001	0.056	0.812	2.587	0.108	0.774	0.379	2.269	0.132	15.049	0.000	2.090	0.148
		PQ	4.744	0.029	53.519	0.000	12.238	0.000	24.406	0.000	23.987	0.000	32.782	0.000	51.411	0.000	23.651	0.000
	Protein-rich	CD	0.677	0.411	13.024	0.000	0.060	0.806	3.730	0.053	1.177	0.278	1.548	0.213	15.361	0.000	2.496	0.114
		PC	0.011	0.916	18.253	0.000	0.219	0.639	6.624	0.010	3.075	0.080	5.731	0.017	22.649	0.000	4.600	0.032
		Qc	8.227	0.004	1.889	0.169	7.575	0.006	0.174	0.677	1.483	0.223	1.124	0.289	3.509	0.061	0.604	0.437
		PQ	16.325	0.000	0.080	0.777	15.788	0.000	2.751	0.097	4.564	0.033	5.527	0.019	0.432	0.511	2.862	0.091

			Bifenthrin															
			Protein free								Protein-rich							
			CD		PC		Qc		PQ		CD		PC		Qc		PQ	
			Chi-Square	Sig.	Chi-Square	Sig.	Chi-Square	Sig.	Chi-Square	Sig.	Chi-Square	Sig.	Chi-Square	Sig.	Chi-Square	Sig.	Chi-Square	Sig.
Pesticide-free	Protein-free	CD	188.529	0.000	173.742	0.000	190.707	0.000	175.647	0.000	170.381	0.000	164.651	0.000	137.677	0.000	100.911	0.000
		PC	255.248	0.000	228.053	0.000	258.405	0.000	246.629	0.000	234.745	0.000	243.088	0.000	221.911	0.000	184.605	0.000
		Qc	206.948	0.000	180.733	0.000	204.962	0.000	188.683	0.000	178.455	0.000	185.881	0.000	165.130	0.000	123.719	0.000
		PQ	227.625	0.000	212.527	0.000	231.681	0.000	217.518	0.000	207.531	0.000	215.057	0.000	187.950	0.000	148.817	0.000
	Protein-rich	CD	209.306	0.000	180.625	0.000	208.857	0.000	193.196	0.000	185.856	0.000	192.487	0.000	172.493	0.000	137.035	0.000
		PC	240.113	0.000	200.397	0.000	238.245	0.000	222.347	0.000	210.643	0.000	223.472	0.000	204.542	0.000	161.619	0.000
		Qc	246.037	0.000	224.427	0.000	246.216	0.000	233.984	0.000	223.490	0.000	230.501	0.000	209.595	0.000	175.921	0.000
		PQ	238.633	0.000	213.746	0.000	241.797	0.000	225.624	0.000	217.239	0.000	219.778	0.000	191.772	0.000	151.442	0.000
Bifenthrin	Protein-free	CD			0.586	0.444	0.446	0.504	2.106	0.147	1.724	0.189	6.129	0.013	11.818	0.001	21.040	0.000
		PC	0.586	0.444			0.039	0.844	0.441	0.507	0.125	0.724	3.369	0.066	7.006	0.008	14.770	0.000
		Qc	0.446	0.504	0.039	0.844			0.820	0.365	0.372	0.542	3.490	0.062	7.738	0.005	17.046	0.000
		PQ	2.106	0.147	0.441	0.507	0.820	0.365			0.092	0.761	0.886	0.347	3.306	0.069	10.722	0.001
	Protein-rich	CD	1.724	0.189	0.125	0.724	0.372	0.542	0.092	0.761			1.680	0.195	4.304	0.038	11.826	0.001
		PC	6.129	0.013	3.369	0.066	3.490	0.062	0.886	0.347	1.680	0.195			1.227	0.268	6.405	0.011
		Qc	11.818	0.001	7.006	0.008	7.738	0.005	3.306	0.069	4.304	0.038	1.227	0.268			2.258	0.133
		PQ	21.040	0.000	14.770	0.000	17.046	0.000	10.722	0.001	11.826	0.001	6.405	0.011	2.258	0.133		
β-cyfluthrin	Protein-free	CD	132.113	0.000	118.011	0.000	131.855	0.000	115.514	0.000	108.033	0.000	107.064	0.000	82.555	0.000	50.931	0.000
		PC	191.131	0.000	178.356	0.000	193.601	0.000	169.091	0.000	158.930	0.000	162.851	0.000	126.564	0.000	79.995	0.000
		Qc	212.722	0.000	187.737	0.000	213.547	0.000	193.656	0.000	191.008	0.000	191.217	0.000	165.401	0.000	127.045	0.000
		PQ	176.162	0.000	161.818	0.000	178.892	0.000	159.939	0.000	149.262	0.000	150.259	0.000	122.607	0.000	83.527	0.000
	Protein-rich	CD	199.668	0.000	181.614	0.000	203.520	0.000	190.421	0.000	181.616	0.000	177.385	0.000	148.707	0.000	114.083	0.000
		PC	209.476	0.000	192.800	0.000	211.405	0.000	193.437	0.000	189.190	0.000	185.084	0.000	156.580	0.000	115.862	0.000
		Qc	234.332	0.000	204.664	0.000	233.796	0.000	219.811	0.000	210.198	0.000	218.888	0.000	198.651	0.000	159.501	0.000
		PQ	246.307	0.000	216.228	0.000	246.096	0.000	231.692	0.000	223.931	0.000	230.643	0.000	213.474	0.000	177.606	0.000

			β-cyfluthrin															
			Protein free								Protein-rich							
			CD		PC		Qc		PQ		CD		PC		Qc		PQ	
			Chi-Square	Sig.	Chi-Square	Sig.	Chi-Square	Sig.	Chi-Square	Sig.	Chi-Square	Sig.	Chi-Square	Sig.	Chi-Square	Sig.	Chi-Square	Sig.
Pesticide-free	Protein-free	CD	14.541	0.000	10.471	0.001	0.801	0.371	4.744	0.029	0.677	0.411	0.011	0.916	8.227	0.004	16.325	0.000
		PC	75.788	0.000	75.760	0.000	11.972	0.001	53.519	0.000	13.024	0.000	18.253	0.000	1.889	0.169	0.080	0.777
		Qc	25.870	0.000	25.998	0.000	0.056	0.812	12.238	0.000	0.060	0.806	0.219	0.639	7.575	0.006	15.788	0.000
		PQ	42.261	0.000	40.075	0.000	2.587	0.108	24.406	0.000	3.730	0.053	6.624	0.010	0.174	0.677	2.751	0.097
	Protein-rich	CD	40.126	0.000	39.183	0.000	0.774	0.379	23.987	0.000	1.177	0.278	3.075	0.080	1.483	0.223	4.564	0.033
		PC	52.899	0.000	58.840	0.000	2.269	0.132	32.782	0.000	1.548	0.213	5.731	0.017	1.124	0.289	5.527	0.019
		Qc	73.267	0.000	72.461	0.000	15.049	0.000	51.411	0.000	15.361	0.000	22.649	0.000	3.509	0.061	0.432	0.511
		PQ	40.470	0.000	36.938	0.000	2.090	0.148	23.651	0.000	2.496	0.114	4.600	0.032	0.604	0.437	2.862	0.091
Bifenthrin	Protein-free	CD	132.113	0.000	191.131	0.000	212.722	0.000	176.162	0.000	199.668	0.000	209.476	0.000	234.332	0.000	246.307	0.000
		PC	118.011	0.000	178.356	0.000	187.737	0.000	161.818	0.000	181.614	0.000	192.800	0.000	204.664	0.000	216.228	0.000
		Qc	131.855	0.000	193.601	0.000	213.547	0.000	178.892	0.000	203.520	0.000	211.405	0.000	233.796	0.000	246.096	0.000
		PQ	115.514	0.000	169.091	0.000	193.656	0.000	159.939	0.000	190.421	0.000	193.437	0.000	219.811	0.000	231.692	0.000
	Protein-rich	CD	108.033	0.000	158.930	0.000	191.008	0.000	149.262	0.000	181.616	0.000	189.190	0.000	210.198	0.000	223.931	0.000
		PC	107.064	0.000	162.851	0.000	191.217	0.000	150.259	0.000	177.385	0.000	185.084	0.000	218.888	0.000	230.643	0.000
		Qc	82.555	0.000	126.564	0.000	165.401	0.000	122.607	0.000	148.707	0.000	156.580	0.000	198.651	0.000	213.474	0.000
		PQ	50.931	0.000	79.995	0.000	127.045	0.000	83.527	0.000	114.083	0.000	115.862	0.000	159.501	0.000	177.606	0.000
β-cyfluthrin	Protein-free	CD			0.556	0.456	26.704	0.000	4.039	0.044	21.744	0.000	17.323	0.000	55.847	0.000	75.238	0.000
		PC	0.556	0.456			24.937	0.000	1.980	0.159	16.859	0.000	14.441	0.000	57.748	0.000	75.492	0.000
		Qc	26.704	0.000	24.937	0.000			13.020	0.000	0.029	0.864	0.764	0.382	5.095	0.024	10.376	0.001
		PQ	4.039	0.044	1.980	0.159	13.020	0.000			9.402	0.002	6.565	0.010	36.417	0.000	52.488	0.000
	Protein-rich	CD	21.744	0.000	16.859	0.000	0.029	0.864	9.402	0.002			0.224	0.636	4.990	0.025	9.979	0.002
		PC	17.323	0.000	14.441	0.000	0.764	0.382	6.565	0.010	0.224	0.636			9.275	0.002	16.272	0.000
		Qc	55.847	0.000	57.748	0.000	5.095	0.024	36.417	0.000	4.990	0.025	9.275	0.002			1.546	0.214
		PQ	75.238	0.000	75.492	0.000	10.376	0.001	52.488	0.000	9.979	0.002	16.272	0.000	1.546	0.214		

© 2017 by the authors; licensee MDPI, Basel, Switzerland. This article is an open access article distributed under the terms and conditions of the Creative Commons by Attribution (CC BY) license (http://creativecommons.org/licenses/by/4.0/).
